# 

**Published:** 1892-05

**Authors:** 


### CONTENTS FOR MAY, 1892.

ORIGINAL COMMUNICATIONS.

The Operative Treatment of Retro-Displacements of the Uterus. By C. C. FREDERICK, B. S., M. D. ... 577
Some Contra-Indications for the Use of Opiates. By MARIE B. WERNER ... 581

CLINICAL REPORT.

A "Much Nervous" Case. By WILLIAM C. KRAUSS, M. D ... 586

TRANSLATION.

Chlorine Water as an Antiseptic in Operations and Injuries of the Eye ... 589

SOCIETY PROCEEDINGS.

Philadelphia County Medical Association ... 593
New York Academy of Medicine—Section on Orthopedic Surgery ... 606

PROGRESS IN MEDICAL SCIENCE.

Medical Jurisprudence ... 616
Medico-Legal and Medico-Ethical ... 619

EDITORIAL.

A National Medical Benevolent Fund ... 623
Current Topics ... 624

PERSONAL.

Dr. E. E. Montgomery—Dr. J. L. Eddy—Dr. A. A. Hubbell—Dr. Wm. C. Krauss—Dr. F. J. Thornbury ... 629

BOOK REVIEWS.

Treatise on Gynecology, Medical and Surgical ... 630
A Manual of Venereal Diseases ... 632
A Manual of Hypodermatic Medication ... 633
Transactions of the Association of American Physicians ... 634
A Practical Manual of Diseases of the Skin ... 635
Diseases of the Bladder and Prostate—A Practical Résumé of Modern Methods Employed in the Treatment of Chronic Articular Osteitis of the Hip ... 636
The Chinese, their Present and Future—The Year-Book of Treatment for 1892 ... 637
Sleep, Insomnia and Hypnotics ... 638
Books Received ... 638

